# Recognition of Modified Conditioning Sounds by Competitively Trained Guinea Pigs

**DOI:** 10.3389/fnbeh.2015.00373

**Published:** 2016-01-26

**Authors:** Hisayuki Ojima, Junsei Horikawa

**Affiliations:** ^1^Cognitive Neurobiology and The Center for Brain Integration Research (CBIR), Graduate School of Medical and Dental Sciences, Tokyo Medical and Dental UniversityTokyo, Japan; ^2^Computer Science and Engineering, Graduate School of Engineering, Toyohashi University of TechnologyToyohashi, Japan

**Keywords:** competitive training, conditioning, recognition of natural sounds, spectral and temporal cues, social interactions, tempo discrimination, guinea pig

## Abstract

The guinea pig (GP) is an often-used species in hearing research. However, behavioral studies are rare, especially in the context of sound recognition, because of difficulties in training these animals. We examined sound recognition in a social competitive setting in order to examine whether this setting could be used as an easy model. Two starved GPs were placed in the same training arena and compelled to compete for food after hearing a conditioning sound (CS), which was a repeat of almost identical sound segments. Through a 2-week intensive training, animals were trained to demonstrate a set of distinct behaviors solely to the CS. Then, each of them was subjected to generalization tests for recognition of sounds that had been modified from the CS in spectral, fine temporal and tempo (i.e., intersegment interval, ISI) dimensions. Results showed that they discriminated between the CS and band-rejected test sounds but had no preference for a particular frequency range for the recognition. In contrast, sounds modified in the fine temporal domain were largely perceived to be in the same category as the CS, except for the test sound generated by fully reversing the CS in time. Animals also discriminated sounds played at different tempos. Test sounds with ISIs shorter than that of the multi-segment CS were discriminated from the CS, while test sounds with ISIs longer than that of the CS segments were not. For the shorter ISIs, most animals initiated apparently positive food-access behavior as they did in response to the CS, but discontinued it during the sound-on period probably because of later recognition of tempo. Interestingly, the population range and mean of the delay time before animals initiated the food-access behavior were very similar among different ISI test sounds. This study, for the first time, demonstrates a wide aspect of sound discrimination abilities of the GP and will provide a way to examine tempo perception mechanisms using this animal species.

## Introduction

A considerable number of studies have used the guinea pig (*Cavia porcellus;* G*P*) as an animal model to study cochlear functions (Prosen et al., 1981; Miller, 2001; Pfingst et al., 2011; Géléoc and Holt, 2014), mechanisms of acoustic trauma (Nicol et al., 1992; Noreña et al., 2010), and learning-induced plasticity of adult auditory cortex (Bakin and Weinberger, 1990; Edeline and Weinberger, 1993; Edeline et al., 1993; Weinberger et al., 1993). More basic functions, such as spatio-temporal representation of acoustic parameters (Taniguchi et al., 1992; Bakin et al., 1996; Horikawa et al., 1996) as well as neural coding of natural sounds, including communication and environment sounds (Suta et al., 2003; Syka et al., 2005; Wallace et al., 2005; Ojima et al., 2010; Grimsley et al., 2011a,b; Gaucher et al., 2013), have also been studied with this animal species. Recently, GPs have been used as potential behavioral models for objective demonstration of a subjective phantom sensation, tinnitus (Dehmel et al., 2012; Berger et al., 2013; Heeringa et al., 2014) and to study behavioral responses to intracochlear electrical stimulation (Chikar et al., 2008; Kang et al., 2010; Agterberg and Versnel, 2014). However, it is traditionally known that training GPs is more difficult than training other rodents (Petersen et al., 1977; Philippens et al., 1992; Agterberg et al., 2010), because GPs tend to freeze to novel stimuli especially when stimuli are aversive. Empirically, even typically trained animals are sometimes unstable in evoking conditioned responses to familiar conditioning stimuli and demonstrate a large variation in their response. This may have led to hesitation of use of the GP in research of the sound discrimination abilities that have been investigated for rats and mice.

Social interactions are well-known to influence animal's behavior (Winslow, 1944; Nakamura et al., 1963; Oldfield-Box, 1967; Scott and McCray, 1967; Stimbert, 1970). For example, competition puts animals in an aggressive state, resulting in altered hormonal levels (e.g., Albert et al., 1988, 1989; van Anders et al., 2011; Cunningham et al., 2012; McCall and Singer, 2012; Carré and Olmstead, 2015) and may drive them to attain more food (Albert et al., 1991). In captive situations in a group, earlier recognition of approaching sounds generated by an animal keeper leads to higher probability of access to food. Thus, social interactions such as competition can raise motivation of competitors during training, and their altered inner state will be memorized for a certain period after training. Based on this perspective, we recently designed a competitive social setting (Ojima et al., 2012) and have successfully trained GPs that had been thought to be less suitable as animal models than rats and mice. In the present study we aim at evaluating their sound discrimination abilities using the social setting protocol developed by Ojima et al. (2012).

Guinea pigs emit 7 to 11 different species-specific calls with distinct types of social behavior associated with them (Arvola, 1974; Berryman, 1976). For example, “purr” calls are made up of a bout of almost identical short noise bursts repeating at approximately equal intervals, and is emitted in conjunction with sexual behaviors like contact seeking (Harper, 1976). Such a social behavior implicates that GPs would potentially recognize sounds with distinct spectral compositions and perceive differences in sound sequence or tempo. However, accumulated data have not been interpreted in conjunction with their sound discrimination abilities, because their sound recognition itself has been rarely investigated from the behavioral view point, except in several studies involving tonal quality or tone-level detection (Heffner et al., 1971; Walloch and Taylor-Spikes, 1976; Petersen et al., 1977; Prosen et al., 1978, 1981; Syka and Popelář, 1980; Harrison et al., 1984; Miller et al., 1995), noise-level detection (Agterberg et al., 2010), and detection of different levels of intracochlear electrical stimulation (Agterberg and Versnel, 2014). Thus, despite potential availability of GPs for research of higher auditory functions, their perception of sounds is mostly unknown except for the perception of unnatural sound bursts.

Unlike pure tones or Gaussian noises, natural sounds that are generated by vibration of objects present in the environment surrounding animal life are generally complex with respect to their spectral composition, fine temporal structure, and rhythm or tempo. These structures are easily learned by animals and humans and underlie the perceptual quality of heard sounds, such as timbre or music (Phillips-Silver et al., 2010). Thus, the primary goal of the present study is to reveal sound discrimination abilities of the GP, especially for natural sounds. For this purpose, we applied the originally developed competition-based training protocol to them, which was less aversive and more effective in driving animals to attend to conditioning sounds (CSs) than other protocols using aversive stimuli. We addressed three questions by modifying the CS in the spectral, fine temporal, and tempo dimensions. First, would a particular frequency range play the dominant role in recognition of natural sounds? Second, would GPs generalize sounds that are slightly different in the fine temporal structure from the original sound? Finally, considering the temporal regularity of component segments in some of their calls, we asked whether GPs could recognize the interval or tempo of repetitive sounds or not.

## Materials and methods

### Animals

The care and use of animals were approved by the animal committee of the Tokyo Medical and Dental University (no. 0150209A and no. 0160311A) and conformed to the National Institutes of Health Guide for the Care and Use of Laboratory Animals (NIH publications No. 80-23, revised in 1996). Guinea pigs (Hartley, SPF, male, body weight of 350–400 g, 5–6 weeks old) were purchased from a commercial supplier (Sankyo Lab., Tokyo) and directly transported to the laboratory. They were experimentally naive, showed no infection of tympanic membrane, and displayed no approaching behavior in response to the CS before reinforcement with food. A few days later, Preyer reflex to abrupt sounds, such as those generated by clapping hands or hitting plastics, was checked for the general hearing ability (Böhmer, 1988). Animals were fasted during the training period (see below for details) but allowed to get access to water freely.

### Training facilities and sound delivery system

Training procedure and facilities were basically the same as those used in a previous study (Ojima et al., 2012) but the total training period was shortened to ~2 weeks. One pair of animals was housed in the same home-cage set in the laboratory (temperature at 23–24°C, lighting from 7 a.m. to 8 p.m.). For daily training, they were temporarily moved to a training arena placed within a sound attenuated chamber that was lined with urethane foam and had a LED light (40W equivalent) on the ceiling. Training arena (W50 × D50 × H30 cm) was made of a sound-absorbing carpet on the floor and metal-mesh walls on all sides, with a custom-made pellet dispenser on the front wall and a water pot on the rear wall. Three video cameras were equipped in the sound-attenuating chamber, one on the ceiling (SH-6C, WTW, Japan) and two near the arena walls (WAT-204CX, WATEC, Japan). In addition, one microphone (F-720, Sony, Japan) and one custom-made infrared motion sensor were placed ~30 cm above the food saucer. The motion sensor, adjusted to detect only quick motions of the head and/or body but not jaw movement such as chewing or gnawing, was used to reinforce visual inspection of the animal's performance (see below). Sound delivery system included a Macintosh computer, an analog equalizer (Q2031B, YAMAHA, Japan), a power amplifier (N220, SONY, Japan), and two identical loudspeakers (NS-10MM, Yamaha, Japan) set 1.7 m above the arena and separated 1 m from each other. The sound delivery system was calibrated at 30 cm above the food saucer using a half-inch condenser microphone (7012, ACO, Japan), and the output from the system was compensated with the 1/3-octave equalizer from 80 Hz (low cutoff) to 12.5 kHz (high cutoff) to keep amplitude fluctuation within ±6 dB at 63 dB SPL. The band-pass range of the system was well within the relatively flat portion of the audiogram previously determined behaviorally for this animal species (see Heffner et al., 1971; Prosen et al., 1978).

### Training sound sets and training procedures

**Figure 1:**
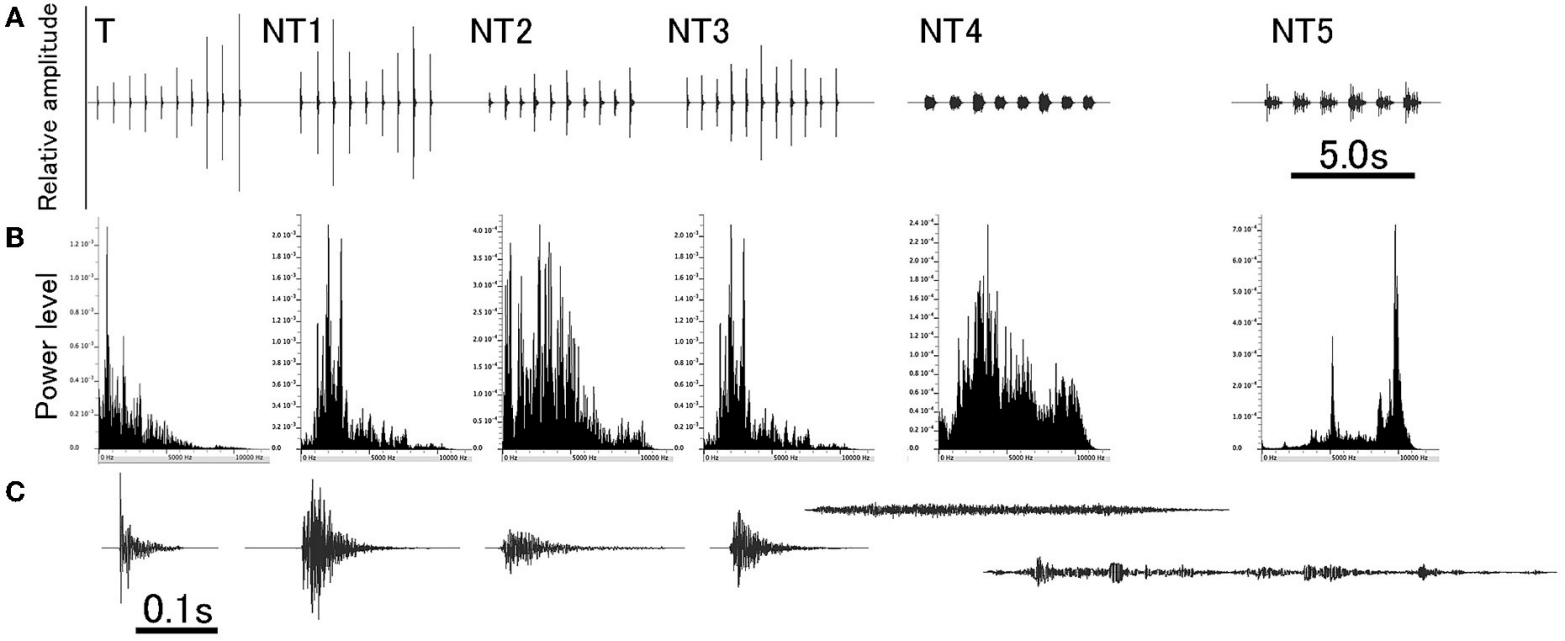
**A set of sounds used for training**. Target (T) sound is a footstep sound and used for conditioning animals (also designated as the conditioning sound, CS). NT1 to NT5 are non-target (NT) sounds of different spectral compositions (NT1, clapping hands; NT2, hitting plastic; NT3, hitting metal; NT4, scratching mesh; and NT5, jingling keys). All sounds are a train of multiple segments that are identical in spectral structure but varied slightly in amplitude. Overall configuration of each of these six different sounds is shown in **(A)**, and the power-spectrum and the enlarged waveform of a single segment from each of these sounds are shown in **(B,C)**, respectively.

Stimuli used in this experiment were derived from natural sounds to which animals were exposed in their home-cage. The sounds were originally generated by stepping on the laboratory floor, clapping hands, hitting a plastic cage, hitting a metal can, scratching a metal mesh, and jingling keys. Being saved as WAV files on a sound editing software (Amadeus Pro, Haire, USA), each natural sound was duplicated several times to make a sequence of the multiple sound segments with an almost identical intersegment interval (ISI). For a given sound sequence, amplitude of individual segments was slightly varied within a range of ± 6 dB. For training, the footstep sound was used as the target (T) sound to condition animals (i.e., the CS) and other sounds as distracting non-target (NT) sounds (Figure 1 and refer to Audio 1). Specifically, the T sound (i.e., CS), 5.7 s long, was comprised of 10 segments, all of which were spectrally identical (ranging from DC up to 12 kHz) with the amplitudes varied slightly. Each segment had duration of 0.08 s (so ISI being 0.63 s) and a damped envelope with more power distributing in the lower frequency region.

One or 2 days (day 1 or day 2) after the transportation (day 0), the preliminary training started. In this stage, animal pairs were frequently fed a small amount of pellets (almost spherical in shape, 4–5 mm in diameter, Sanko, Japan) roughly in synchrony with each playback of the CS (see below for details) through a dynamic speaker (NS-10MM, Yamaha, Japan) placed ~1 m distant from the home-cage front wall. Diet was strictly controlled during this stage by weighing the animal 2–3 times per day so that their body weight was maintained about the 90% level of that on the day of transportation. If the body weight of one of the pair was severely reduced, probably because of the dominant-subordinate relationship, feeding was individually adjusted to balance body weight between the paired animals.

Competitive training was carried out at two stages extending for ~1 week. One pair of cage-mates was placed in the same training arena within the sound-attenuating chamber to make compete for access to food. Animals were trained to discriminate the T from NT sounds for several days (early training stage, ~3–4 days). Thereafter, each animal of the pair was separately trained in otherwise the same way as the early training stage (late training stage, ~2–3 days). Over the following 2 days, the animals were individually subjected to three different types of test trials (see below for details) for behavioral evaluation. Throughout these training stages, pellets were automatically given at a constant delay of 1.6 or 3.2 s (depending on animals) after the T sound termination. The animals were fed additional amounts of pellets in their home-cage between sessions as well as after the daily training so that they gradually gained weight day after day during these stages.

One training session consisted of six trials each of which contained 1 CS as a target (i.e., T sound) and 5 NT sounds as distractors. For the daily training, 5–6 sessions were given to each pair of animals and 2–3 sessions to each animal of the pair during the early and late training stages, respectively. These sounds were played at various inter-sound intervals ranging from 66 to 132 s with their order randomized based on the trial, session, day and animal.

### Test sound sets and test procedures

Test sounds were generated by digitally modifying CS segments using the sound editing software either in the spectral, fine temporal or ISI (i.e., tempo) domain, and were designated as pseudo-target (PsT) test sounds.

**Figure 2:**
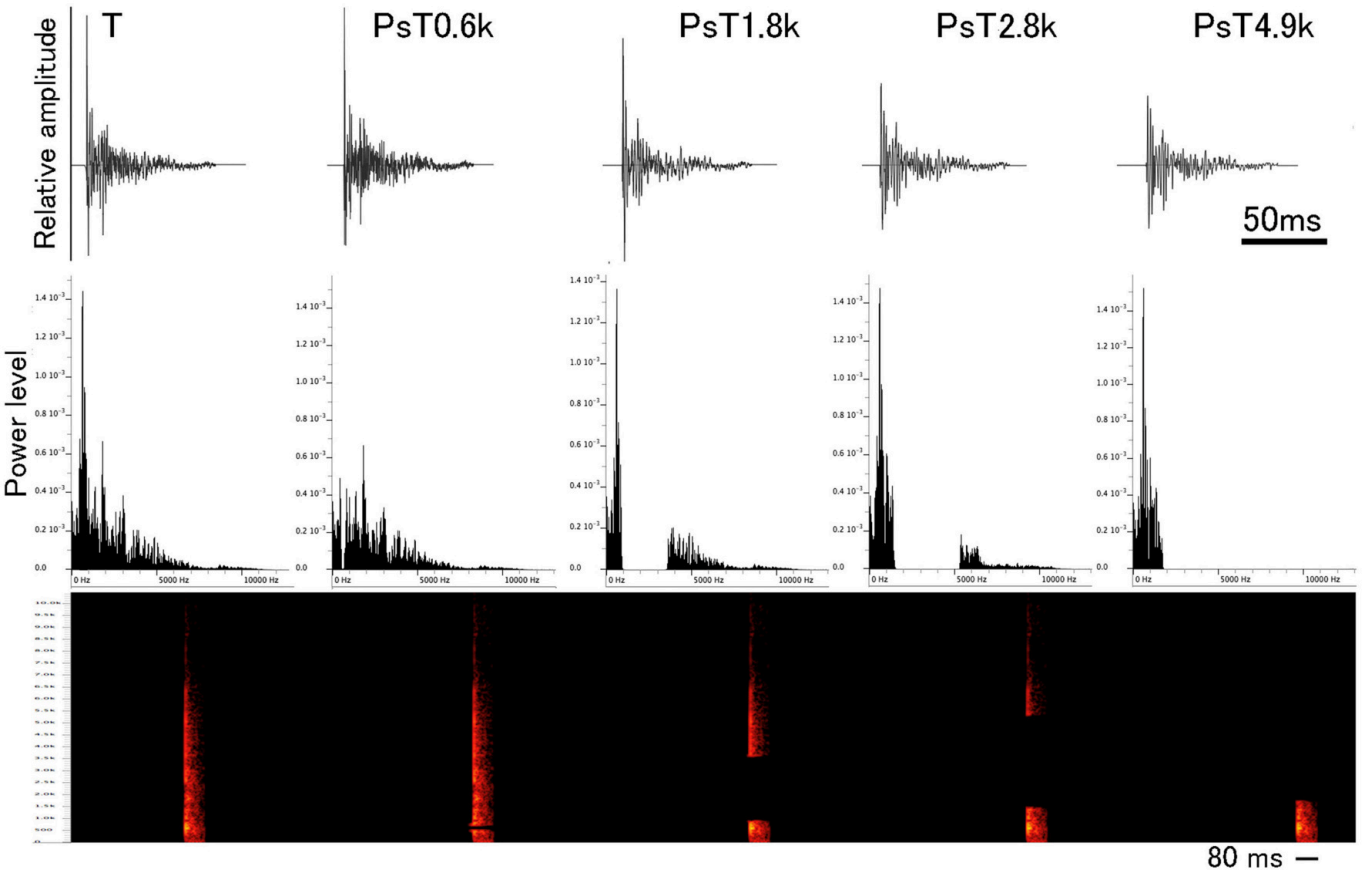
**Waveforms and power spectra of single segments from the conditioning sound (designated as the T sound) and four test sounds used in the spectral modification test**. Below waveform, sonogram is also presented. To generate the spectral modification test sounds, frequency ranges centered at 0.6, 1.8, 2.8, and 4.9 kHz are separately eliminated from the CS (see this Figure for their power spectra).

For spectrally modified PsT test sounds, four different frequency ranges were separately filtered out from the individual CS segments (Figure 2, also refer to the 1st sound of Audio 2) in such a way that the energy (RMS unit) of the eliminated frequency ranges was kept constant among different test sounds (1.8 ± 0.1 dB, mean ± S.D.). This filtering-out was centered at each of the 0.6 kHz (ranging 0.50 – 0.72 kHz), 1.8 kHz (ranging 0.90 – 3.60 kHz), 2.8 kHz (ranging 1.48 – 5.3 kHz), and 4.9 kHz (ranging 1.75 – 12.0 kHz), which corresponded to major energy peaks of the sound segments (refer to the 2nd, 3rd, 4th, and 5th sounds of Audio 2, respectively). Before reproducing to animals, the overall energy levels of PsT test sounds were equalized to that of the T sound. Using pairs of the PsT and T sounds, we assessed the spectral-range preference of the animals in recognition of the CS.

**Figure 3:**
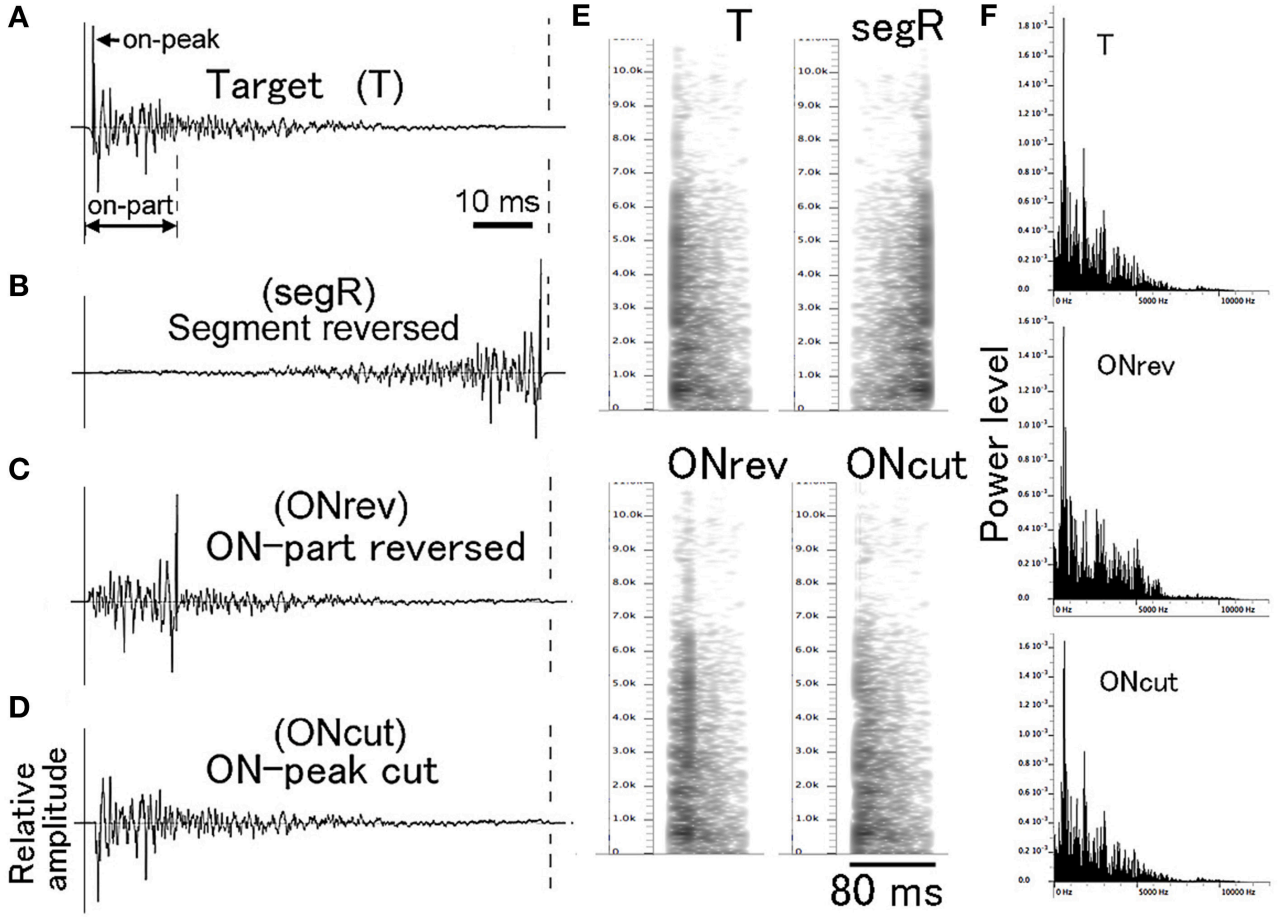
**Waveforms (A–D), sonograms (E), and power spectra (F) of single segments used in the fine temporal modification test**. Target (T) sound is used as the conditioning sound (CS). ONrev is a test sound modified from the T sound by reversing its early 17-ms portion (on-part) and ONcut is a test sound modified from the T sound by eliminating its 1-ms onset attack portion (on-peak).

For PsT test sounds modified in fine temporal structure, individual CS (i.e., T sound) segments (Figure 3A) were manipulated along the time axis in three different manners (Figures 3B–D). Using this type of test sounds, we aimed at examining how important the temporal integrity of the CS was for its recognition. For the full disturbance of temporal integrity, the individual segments of a multi-segment CS were time-reversed without changing their order (segR; refer to the 2nd sound of Audio 3). For partial disturbance of the temporal integrity, only the early 17-ms portions of the individual CS segments were locally reversed in time (ONrev; refer to the 3rd sound of Audio 3). Furthermore, it was examined how important the onset transient was for recognition of the CS. The attack portion with the maximum amplitude (1 ms in duration) was cut out from the individual CS segments (ONcut; refer to the 4th sound of Audio 3). Before exposing to animals, the overall energy level of these test sounds was equalized to that of the T sound. It is noted that these fine temporal modifications affect not only temporal integrity but also spectral structure to some extent. However, on the power spectrum inspections, such temporal alterations affected the spectral structure very little (compare T and ONcut in Figure 3E) or to some extent (compare T and ONrev in Figure 3E). It is also known that the spectral structure of pair of forward and reversed sounds is identical in the long-term FFT analysis (Patterson, 1994; Lu et al., 2001). Thus, we believe that these modifications would disrupt predominantly the temporal integrity of the T sound.

**Figure 4:**
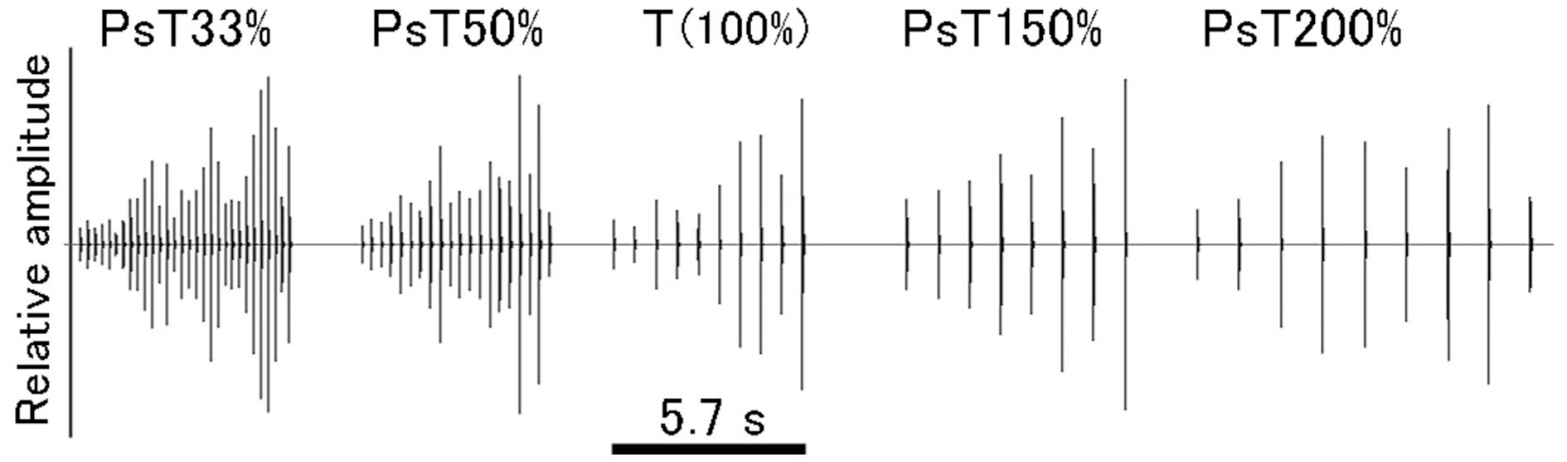
**Overall configuration of each of the test sounds (or pseudo-target, PsT, sounds) used in the tempo modification test**. Test sounds have the intersegment interval (ISI) either decreased to 33 or 50% or increased to 150 or 200% of the original ISI of the CS (100%, 0.63 s).

Finally, to generate tempo-modified PsT test sounds, the ISI of CS segments was reduced either to 33 or 50% or increased either to 150 or 200% of that of the CS segments (100%). The overall sound duration of these PsT test sounds was adjusted to a relatively constant range (7.3 ± 1.9 s, mean ± S.D.) by changing the number of segments per test sound (Figure 4; also refer to Audio 4).

Each test sound was added to a single training sound set to make a test sound set (i.e., including 1 PsT, 1 T, and 5 NT sounds). Test sound sets were reproduced to individual animals over 2 days following the 2-week training period. Test sounds were played at various inter-sound intervals ranging from 66 to 132 s in order randomized on the trial, session, day, and animal bases.

### Behavioral evaluation

In each session, sound waveforms in source files, sounds monitored by the in-chamber microphone, timings of the feeding, and timings of head motions were continuously recorded on a single chart of SPIKE 2 software via an A-D converter (Micro 1401 mkII, CED, England). The same chart and the video images taken at three different angles were also saved together in a DVCAM recorder (DSR-45A, SONY) to ensure synchronization between sound and behavior and used for off-line analyses (see lower-bottom panel of Videos 1, 2, 3).

By viewing the animal's behavior and the timings of sound onset/offset as well as the feeding timing on the DVCAM videos, animal's performance was evaluated by authors and in part by two female students (about 96% agreement rate between the two groups). Distinct behavioral reactions (BhRs; see Results and Video 1), which were easily discriminated from spontaneous food-access motion, were regarded as positive response signs. Trials were considered to be positive only if the following two criteria were fulfilled; (1) animals initiated the BhRs during the sound-on period and (2) they continued these BhRs at least for 3.2 s after the sound termination. This time corresponds to the time interval between the sound termination and the feeding time in the T sound trials. We assume that animals have perceived test sounds to be the same or in the same category as the T sound, if they continue the once-initiated BhRs until the end of this 3.2-s period. Note that trials were considered to be negative (i.e., discriminative) when animals discontinued the once-initiated BhRs within the sound-on period or the 3.2-s period. In this case, we assume that animals have perceived test sounds to be different from the T sound.

Animals were able to move freely during experiment, but mostly stayed near the food saucer except for consumption of water located across the training arena. Reaction time (RT), which was defined as the time from the sound onset to the moment when animals initiated the food-access motions, regardless of their behavioral consequences thereafter, was measured by scanning the video images in a frame-by-frame manner (33 ms per frame). Measurements were rounded off to two decimal places.

### Statistics

In each modification test type, Cochran's *Q*-test was used for non-parametric comparison among the different test sounds, and was followed by McNemar's test adjusted by Bonferroni correction for multiple comparisons between different PsT test sounds (**Figures 6–8**). For non-parametric comparison between T and PsT sounds, McNemar's test was used (**Figures 6–8**). One-way repeated-measures ANOVA was used for parametric comparison among the RTs to sounds with different tempos (**Figure 9**). Finally, paired Student's *t*-test was applied for comparison of the RTs between the T sound and the tempo-modified PsT test sounds (**Figure 9**).

## Results

### Social behavior during the competitive training

**Figure 5:**
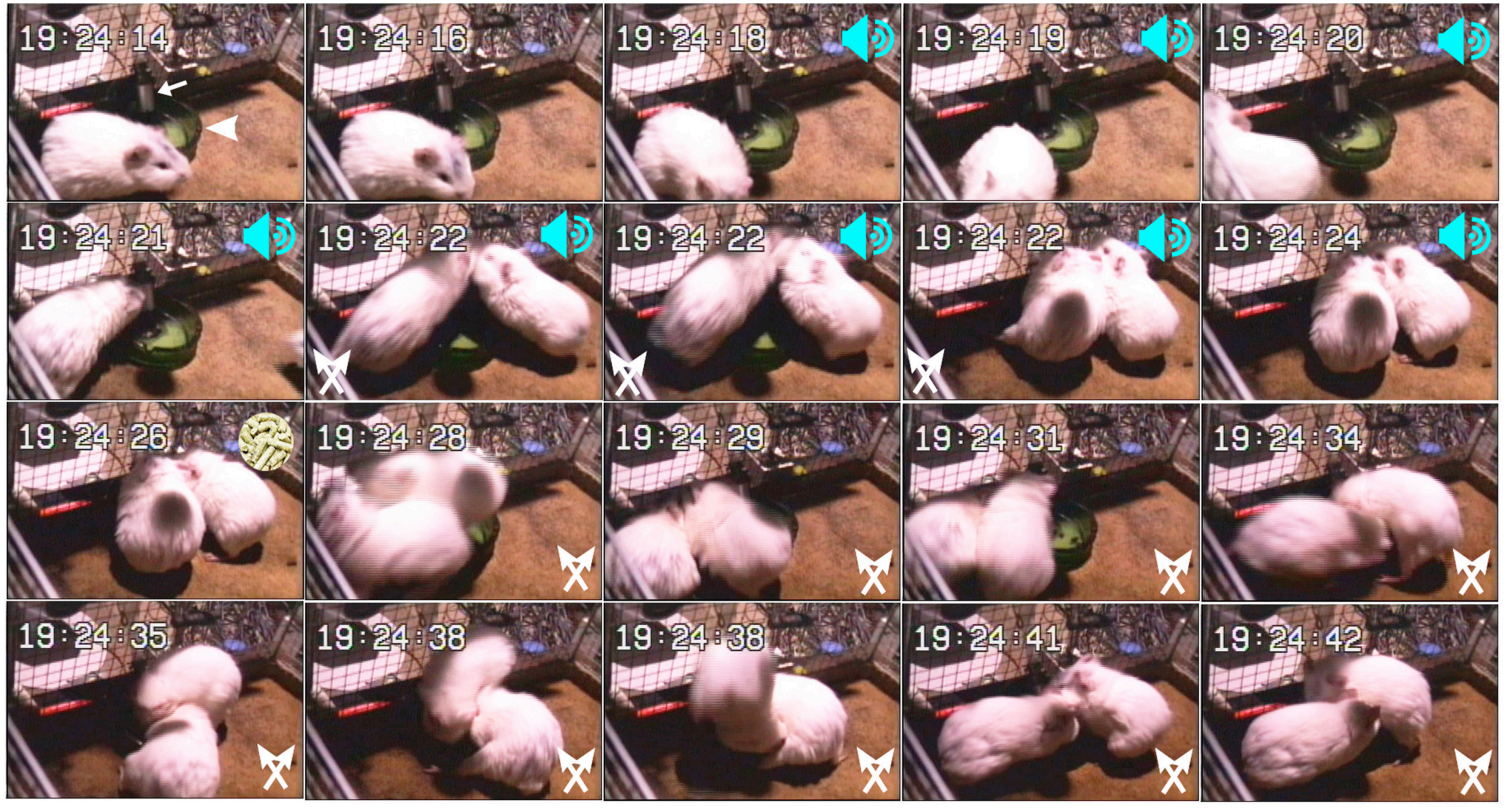
**Sequence of video frames showing conflicts typically observed soon after the onset of the conditioning sound (CS) during the competitive training**. A pair of two guinea pigs (GPs) has been trained for 10 days. During the sound-on period (indicated by speaker symbols), footstep sound (CS) is reproduced, and 1.6 s after the sound termination, food is given as reinforcement (indicated by a pellet photo). Frames with crossed arrows indicate the conflict behavior between the two competitors. Also see Video 1.

Thirty GPs were subjected to training: six for the tests of all three types, 12 for the spectral and fine temporal tests, and the remaining 12 for the tempo test alone. Usually on the day 1 or day 2, starved GPs started to spontaneously emit a whistle call (Berryman, 1976) to demand food. When this demanding call was consistently emitted, they frequently showed approaching behavior to the food saucer in response to the CS. It was common to observe conflict behaviors such as keeping their body over the food saucer to block the competitor's approach to it and/or inserting their snout at the orifice of the food hopper to interfere with competitor's food intake (Figure 5 and Video 1).

### Behavioral reactions to the target (T) and non-target (NT) sounds after conditioning

In the early training stage, paired GPs competed for food with the conflict behavior shown above and also initiated distinct food-approaching behaviors including quick head swaying combined with neck extending above the food saucer and/or circling about it (i.e., BhRs; see the 1st scene of Video 2) at the onset of the T sound. Although some animals evoked both modes of the BhRs, most evoked only either of them, usually head swaying more frequently. These BhRs were more consistently evoked in the later training stage in which animals were separately trained. Spontaneous behavior at the food saucer was easily discriminated from these BhRs mainly on the basis of quickness and business. In contrast, from the very beginning of the early training stage, animals came to ignore the NT sounds (see the 3rd scene of Video 2), resulting in virtually no false positive response (i.e., no BhR) to these sounds. Three sessions immediately before the test trials showed that all animals securely evoked the BhRs almost exclusively to the T sound, with rare miss-responses to the T sound (< 5% per animal, on average) as well as very rare false positive responses to the NT sounds (< 1% per animal, on average).

### Behavioral reactions in the three generalization tests

#### Spectral modification test

**Figure 6:**
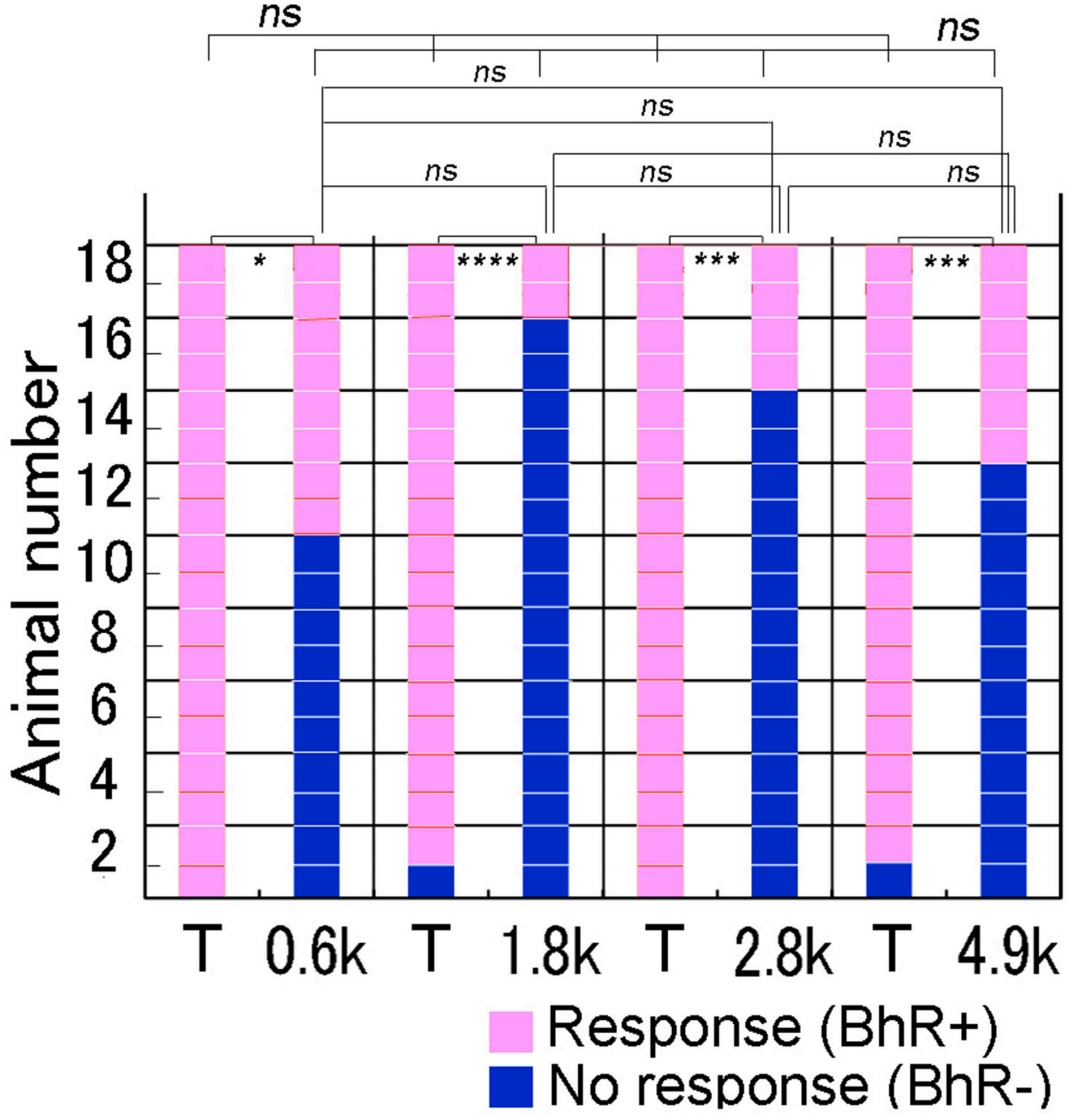
**Performance of individual animals tested for discrimination of spectrally modified test sounds**. In a test trial, one test sound is reproduced to animals together with the T sound (i.e., CS) as the positive control (see abscissa). Behavioral responses of each animal (on the ordinate) to the T and test sounds are shown along the horizontal line, while behavioral responses of all animals to each sound (on the abscissa) are shown in the vertical column. Animal's responses are considered to be positive (in pink) when they evoke unique behavioral reactions (BhRs, see text for details) that continue for a 3.2 s after the sound termination. When BhRs are not evoked or broken off, animal's behavior is considered to be negative (in dark blue). We suppose that animals initiate and continue the BhRs, because they have perceived the modified test sound to be the same as the CS, while animals discontinue the BhRs, because they have perceived the test sounds to be different from the T sound (i.e., CS). ^#^*p* < 0.05; ^*^p < 0.005; ^**^p < 0.001; ^***^p < 0.0005, ^****^p < 0.0001, and ns: *p* > 0.05. The caption is also applicable to Figures 7,8.

Single animals were given each of the four different spectral modification test sounds once. As shown in Figure 6, when the test sound was lacking a frequency band centered at 0.6 kHz (see Figure 2), several of 18 GPs (8/18) initiated the BhRs as positive responses, indicating that they perceived this test sound to be in the same category as the CS. The remaining 10 subjects showed no BhRs or initiated but discontinued them before the sound termination, indicating that they perceived this test sound to be different from the CS. In contrast, when the T sound was played as the positive control to the same animals, all the animals (18/18) responded positively to the T sound with the typical BhRs. The ratio of the number of animals responding positively relative to the number of animals used (positive animal ratio) was significantly different (*p* < 0.005) between the two sound groups. Similarly, to the 4.9 kHz-centered band-rejected test sound, several of the animals (6/18) responded positively, while virtually all the animals (17/18) responded positively to the T sound (*p* < 0.0005). A similar tendency was observed for the test sounds having frequency ranges eliminated more centrally, with the positive animal ratios slightly smaller than those for the frequency ranges centered at the 0.6 and 4.9-kHz. Namely, to the 1.8 and 3.0-kHz-centered band-rejected test sounds, only a few animals (2/18 and 4/18) responded positively, while virtually all of them (17/18 and 18/18) did to the T sound played together (*p* < 0.0001 and *p* < 0.001, respectively). These results indicate that the animals could discriminate between the spectrally modified test sounds and the T sound. However, the positive animal ratios for the four test sounds were not statistically significant (Cochran's *Q*-test, *p* = 0.064), suggesting that there may not be a preferred frequency range for recognition of the CS by GPs.

#### Fine temporal modification test

**Figure 7:**
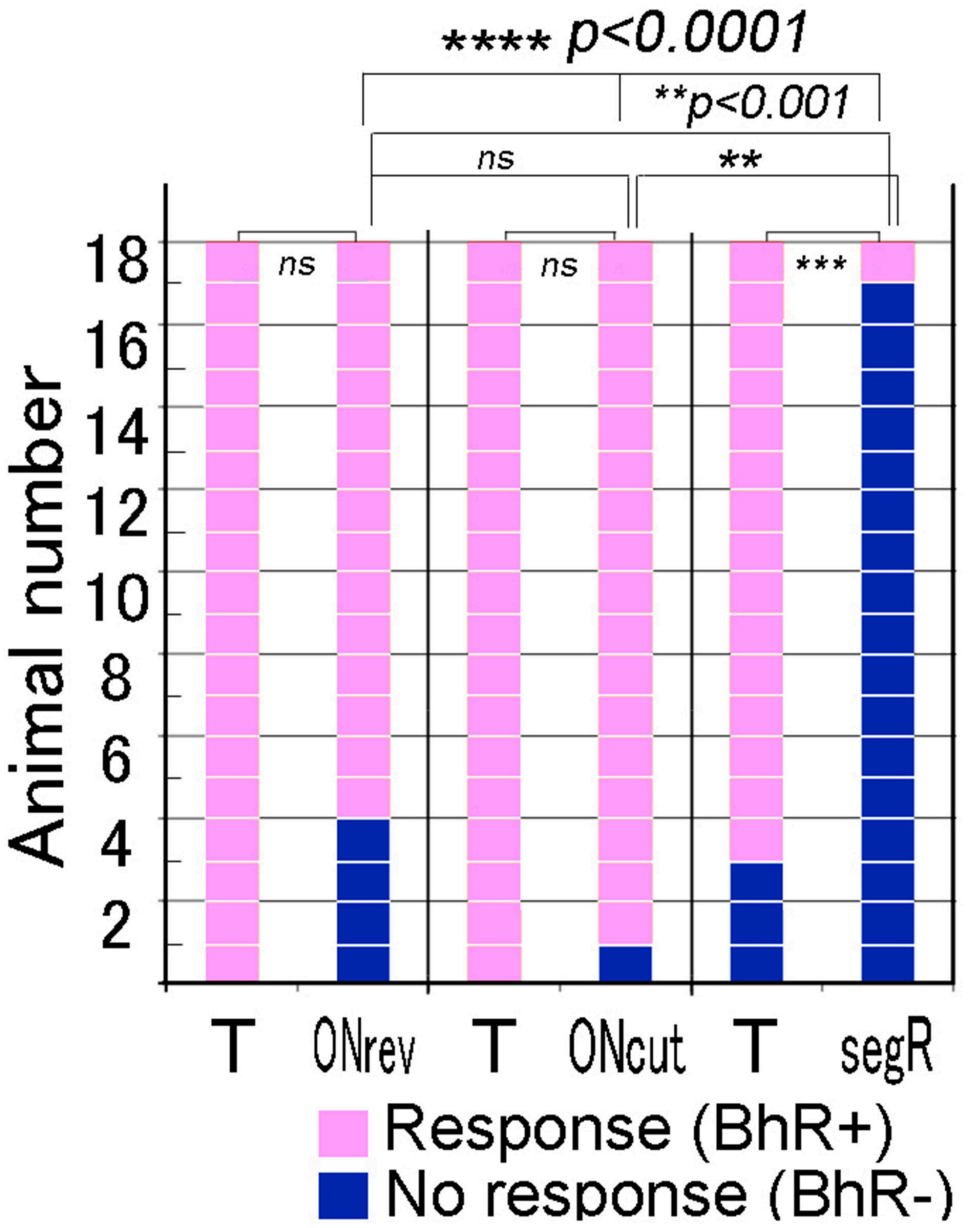
**Performance of individual animals tested for discrimination of the test sounds modified in fine temporal structure**. For the temporal test sounds, temporal structure of the CS is disturbed fully (segR), partially (ONrev) or only at the onset attack portion (ONcut). The target (T) sound (i.e., CS) is also played within each test trial and used as the positive control. Squares in pink indicate positive reactions, while those in dark blue indicate no reaction. ^#^*p* < 0.05; ^*^p < 0.005; ^**^p < 0.001; ^***^p < 0.0005, ^****^p < 0.0001, and ns: *p* > 0.05.

Single animals were given each of the three different temporal modification test sounds once. The positive animal ratios were significantly different among these test sounds (Cochran's *Q*-test, *p* < 0.0001). As shown in Figure 7, when the 80-ms segR test sound was played to 18 animals, only one animal (1/18) displayed the BhRs (see the 2nd scene of Video 2), while most of them (15/18) responded with the typical BhRs to the T sound played as the positive control (*p* < 0.0005), indicating that the animals perceive the segR sound to be different from the CS. In contrast, when the test sound generated by time-reversing the 17-ms ON-part of the T sound segments (ONrev) was played, most animals (14/18) initiated the typical BhRs, while all the animals responded positively to the T sound played as the positive control (18 of 18; statistically not significant). Virtually all animals (17/18) responded positively to the test sound lacking the 1-ms onset transient peak (ONcut) as consistently as they did to the T sound played as the positive control (statistically not significant).

#### Intersegment interval modification test

**Figure 8:**
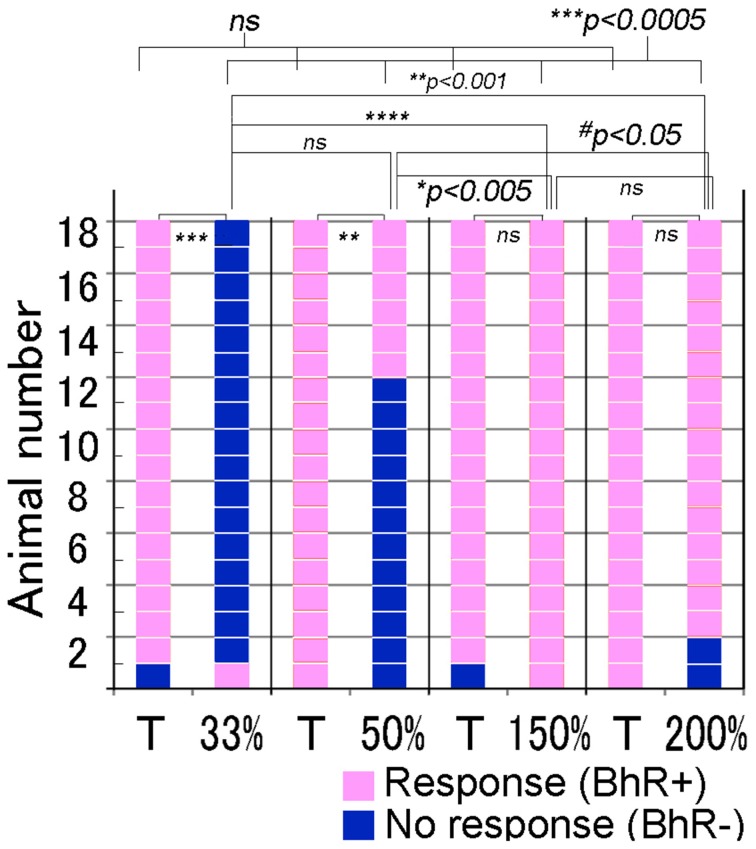
**Performance of individual animals tested for discrimination of the test sounds modified in tempo or intersegment interval (ISI)**. To make the tempo test sounds (indicated in %-ISI), the ISI of the target (T) sound (i.e., CS) is shortened or elongated to the duration indicated in %. Target (T) sound is also played as the positive control together with the test sound in the same test trials. *Pink* indicates the positive response, while *dark blue* indicates the negative, so discriminative, response. ^#^*p* < 0.05; ^*^p < 0.005; ^**^p < 0.001; ^***^p < 0.0005, ^****^p < 0.0001, and ns: *p* > 0.05.

**Figure 9:**
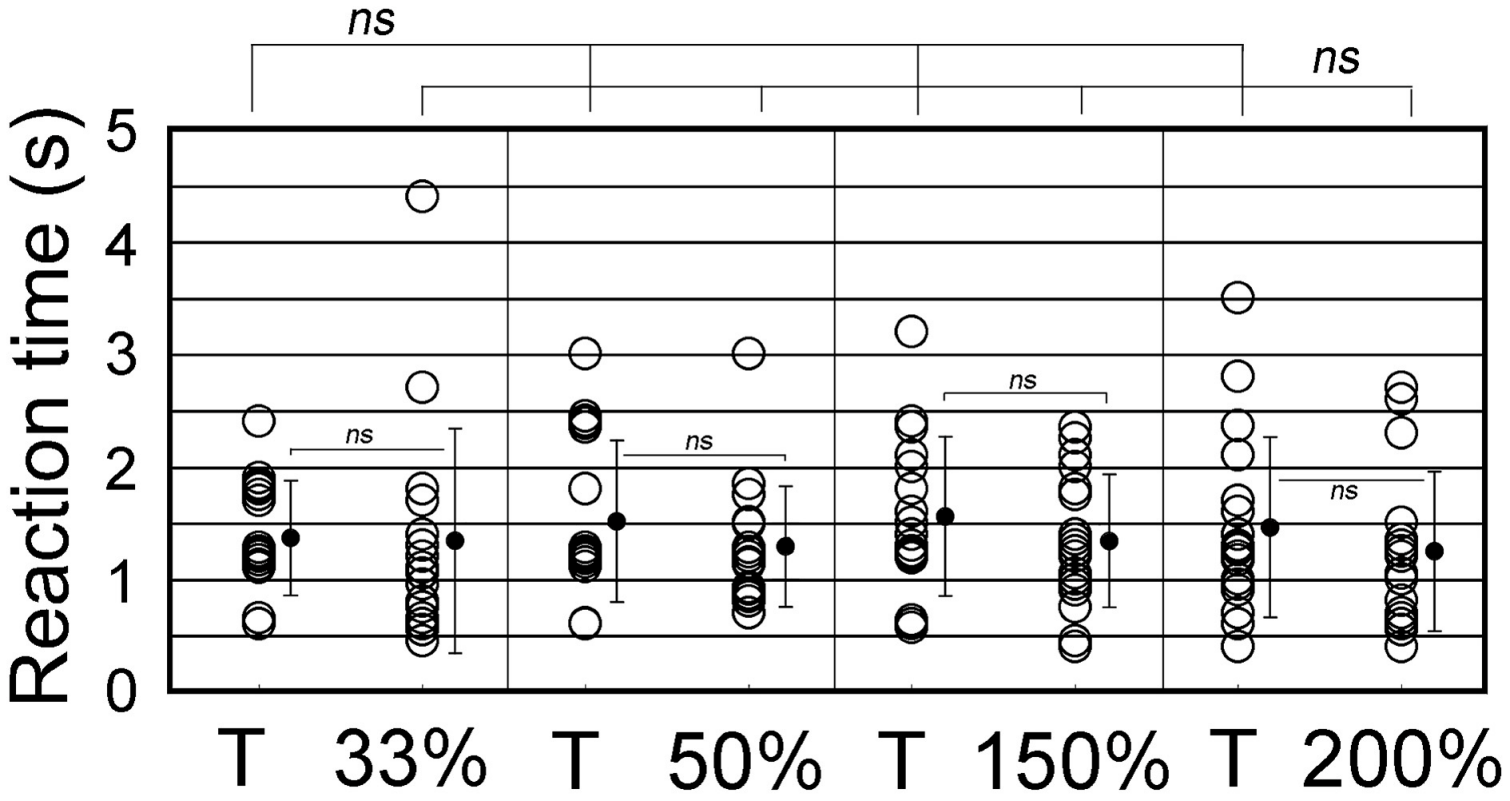
**Reaction time (RT) measured from the sound onset to the time when animals initiate the food-approaching motions (see text for detail)**. The ISI of T sound (100%) is increased to either 150 or 200% or decreased to 33 or 50% to generate tempo-modification test sounds. Target (T) sound (i.e., CS) is also played as the positive control together with the test sounds. Circles represent individual animals. When more than one trial takes the same RT, their data points are slightly shifted vertically for display purpose. Vertical lines indicate standard deviation.

We further examined whether animals responded differently to ISI- or tempo-modified test sounds (see Video 3). In the tempo discrimination test, test sounds had the ISI changed to 33, 50, 150, or 200% of the original ISI of the CS (100%, 0.63 s). The positive animal ratios were significantly different among these ISI test sounds (Cochran's *Q*-test, *p* < 0.0005). As shown in Figure 8, none of the subjects but one (1/18) displayed the positive behavior in response to the 33%-ISI test sound (see the 3rd scene of Video 3), strongly contrasting the nearly perfect positive responses to the T sound played as positive control within the same trials (17/18, see Video 3; statistically significant, *p* < 0.0005; Figure 8). In more detail, two of them did not move at all during the presentation of the 33%-ISI test sound, while the remaining 15 animals either evoked instantaneous motions such as bobbing head for a while (*n* = 13) or initiated but discontinued the BhRs within the sound-on plus 3.2-s period (*n* = 2). The mean RT measured from the sound onset to the motion onset was 1.34 ± 1.0 s (S.D.), ranging from 0.5 to 4.4 s (*n* = 16, one positive response included) and was not significantly different (*p* = 0.77) from the RT for the T sound played within the same trials (1.38 ± 0.59 s, mean ± S.D., ranging from 0.6 to 2.4 s, *n* = 17; Figure 9).

To the test sounds with the ISI reduced to 50%, 6 out of 18 subjects showed the positive behavior, and the remaining 12 animals were evaluated to be negative (so discriminative), including eight that initiated only the instantaneous food-access motions and four that discontinued once-initiated BhRs during the sound-on plus 3.2-s period, although all 18 animals displayed the typical BhRs to the T sound played as the positive control (statistically significant, *p* < 0.001; Figure 8). The mean RT for the test sounds was 1.29 ± 0.54 s (mean ± S.D., ranging from 0.7 to 3.0 s, *n* = 18, positive responses included), and statistically not different (*p* = 0.28) from that of the T sound (1.53 ± 0.72 s, mean ± S.D., ranging from 0.6 to 3.0 s, *n* = 18; Figure 9).

In contrast, when the test ISI was increased to 150 or 200% the original ISI, virtually all GPs (18/18 and 16/18) responded to them with the typical BhRs (see the 2nd scene of Video 3) as consistently as they responded positively to the T sound played as the positive control (17/18 and 18/18, respectively; statistically not significant; Figure 8), indicating that they ignored the ISI changes. The mean RT was 1.34 ± 0.59 s (S.D., ranging from 0.4 to 2.3 s, *n* = 18) for the 150%-ISI test sounds and 1.25 ± 0.78 s (S.D., ranging from 0.4 to 2.7 s, *n* = 16) for the 200%-ISI test sounds (Figure 9). These values were similar to the RT for the respective T sound played as the positive control (1.55 ± 0.78 s, mean ± S.D., ranging from 0.6 to 3.2 s, *n* = 17 in the 150%-ISI test trials and 1.45 ± 0.80 s, mean ± S.D., ranging from 0.4 to 3.5 s, *n* = 18 in the 200%-ISI test trials). Neither pair of the T and 150%-ISI test sounds nor pair of the T and 200%-ISI test sounds was statistically significant (*p* = 0.35 and 0.41, respectively).

## Discussion

### Possible mechanisms of sound learning in cortex

The present study shows that GPs can recognize natural sounds on the basis of their spectral and temporal structures and that such perceptual capabilities are learned in the classical conditioning procedure using food as positive reinforcement. The CS used was a set of noise-like footstep sounds, which had distinct energy peaks at several frequencies, typical of natural sounds. Previous studies showed that tones used as the CS could be memorized in the spectral (Weinberger et al., 1984; Recanzone et al., 1993; Kilgard and Merzenich, 1998a) and probably temporal (Kilgard and Merzenich, 1998b; Beitel et al., 2003) dimensions directly in auditory cortex, including the primary field. However, how complex sounds, such as our natural sounds, are memorized in the auditory cortex is hardly known. The above mentioned studies revealed changes in spectral representation of neuronal populations and, more globally, remodeling of tonotopic maps, such as expansion of the domain representing the frequency of a tone used as the CS (Recanzone et al., 1993; Bao et al., 2001) and suggested that such cortical plasticity would underlie tone discrimination abilities. Since our CS used has a noise-like spectral dispersion, the expansion of cortical regions representing particular frequencies would not be expected. Instead, it is expected that the region representing lower frequencies to which the majority of energy of the CS is assigned, so the more anterior portion of the primary auditory field of the GP (Redies et al., 1989), might be expanded. Future studies comparing patterns of cortical activation using electrophysiological measurements and/or optical imaging techniques between naive and trained subjects would promise to reveal such a cortical remodeling established during training.

Guinea pigs are social as exemplified by previous behavioral observations, such as “frequent conflict among cage-mates for access to food” (Harper, 1976). It is traditionally known that training of GPs is difficult or unstable. We frequently observed in preliminary trials that if trained in isolation, they tended to be less motivated, even if starved. Alternatively, they sometimes failed to be conditioned to the CS but were conditioned to feeding-associated noises or to sounds generated when pellets fell into the food saucer. In the present training, competition is likely to raise motivation of competitors and drive them to attend earlier to the cues predicting food, since the winner can take all at a higher probability, if it initiates approaching for food earlier and faster than the looser. Such competition-driven behavioral pattern must have been learned and stored as memory in association with stimulus sounds in the neural circuitry involving the primary auditory field (Weinberger, 2004).

### Recognition of sounds modified in spectral structure

In the present study, we generated spectral test sounds by eliminating different frequency ranges with their energy kept constant. These PsT test sounds were presented to animals after adjusting the overall energy level to that of the positive control sound (i.e., T sound). All of these PsT test sounds were almost equally discriminated from the T sound (see Figure 6; Cochran's *Q*-test). In our previous study (Ojima et al., 2012), different frequency ranges were eliminated from the CS to make spectral modification test sounds like in the present study, but the energy of these eliminated ranges was also varied among the test sounds. Results showed that discrimination performance to these test sounds was roughly proportional to the amount of energy eliminated. Indeed, test sounds that had the 20, 26, and 55% energy eliminated from the CS were discriminated by 31, 46, and 85% of animals tested, respectively. Therefore, two parameters were thought to be responsible for the discrimination behavior, one being the spectral range and the other being the amount of energy. Considering the present results suggesting that the discrimination behavior of GPs does not rely on frequency ranges, our previous results can be interpreted as that the difference in discrimination was ascribed to the difference in the energy amount eliminated.

### Recognition of sounds modified in fine temporal structure

When animals recognize the CS, there is a possibility that they might use the onset attack portion as a temporal cue, since this portion generates a click-like percept, if it is played in isolation. When the ONcut (elimination of the 1-ms onset portion from the CS segments) test sound was presented to animals, they appeared not to care about such a small difference (Figure 7). For this test sound, the temporal structure was transiently disintegrated at the sound onset but, as indicated by the comparison of its power spectra with that of the T sound (see Figure 3E), these two spectra had almost identical envelope shapes, although the power of the modified sound was slightly reduced. Thus, to the extent of the present temporal modification, it is likely that the animals relied predominantly on the overall spectral composition for recognition of the CS and that their recognition was not deteriorated either by the transient (i.e., 1-ms) disturbance in temporal structure or changes in the overall energy level. This is consistent with our previous data in which test sounds modified from the CS only in intensity were not discriminated by GPs (Ojima et al., 2012).

Then, to see substantial modification effects, we dramatically changed the temporal structure by time-reversing the entire duration of the CS segment (segR) without changing their order. This segR test sound was almost completely discriminated from the T sound used as the positive control (Figure 7). Since the long-term spectral compositions of such a sound pair are identical (Patterson, 1994; Lu et al., 2001), it is suggested that the overall temporal structure is critical for recognition of this sound. When the segR and ONcut test sounds were compared, the time-reversed portion of the former was 80 ms long, while that of the latter was only 1 ms long, and the discrimination of the former was almost complete, while that of the latter was very poor. Accordingly, we examined the effect of the reversal of intermediate duration on the animal's perception by time-reversing the early 17-ms portion of the CS segment (roughly 50% energy included, ONrev; see Figure 3C). Animals' behavior was not significantly affected by this considerable disintegration in temporal structure (Figure 7). This is compatible with human psychophysical experiments in which time-reversing of short-term segments generated by subdividing single speech sentences did not affect intelligibility of these modified sentences, even if the segment duration was as long as 50 ms (Saberi and Perrott, 1999). We assume that the limen for GPs to detect the disintegration in temporal structure of the CS is larger than this 17-ms duration but smaller than the 80-ms full duration of the CS segment.

### Recognition of sounds modified in tempo

Rhythm is one of the critical components for speech processing, motor coordination, and music perception. Humans can easily discriminate rhythmic and unrythmic tone patterns (Hulse et al., 1984a) as well as different rhythms of repeated tone sequences (Hulse and Kline, 1993). Rhythm can be reduced to a simpler form of temporal component, tempo. Classical studies have shown that a variety of animals can perceive tempos, including European starling (Hulse et al., 1984a,b), quails (Schneider and Lickliter, 2009), pigeons (Farthing and Hearst, 1974; Hagmann and Cook, 2010), rats (Mostofsky et al., 1964; Crites et al., 1967; Weiss and Schindler, 1981; Meck et al., 1985, 2013), cats (Dong et al., 2011), and non-human primates (McDermott and Hauser, 2007). In generalization testing after tempo discrimination training, it was classically claimed that the distribution pattern of correct responses across different tempo values (i.e., discrimination gradient) were determined by whether reinforcement was *differentially* or *non-differentially* assigned to tempos used for conditioning. For rats and pigeons, *differential* discrimination training, in which one of two different click rates was reinforced but the other was not, led to the discrimination gradient with a progressively reducing performance bilaterally away from the positive peak located at the reinforced tempo. On the contrary, *non-differential* discrimination training, in which periods of a click rate were reinforced but the intervening silent periods were not, led to a relatively uniform discrimination gradient (Mostofsky et al., 1964; Weiss and Schindler, 1981). In our tempo modification tests of the non-differential type, we showed that discrimination performance to test tempos was asymmetrical along the tempo gradient; that is, good discrimination to tempos faster than the reinforced one vs. virtually no discrimination to tempos slower than the reinforced one. This asymmetrical performance is incompatible with either of the response patterns just mentioned above. Consequently, we will discuss possible mechanisms of the tempo discrimination by GPs from a different point of view and intend to interpret it comprehensibly on the basis of a relationship between the temporal integration time window (TITW) and the time intervening between repeated sounds.

TITW is a basic psychological concept in sound perception in human (Bregman, 1990; Moore, 2003; Grondin, 2010; Grahn, 2012) and in animals, mostly in monkeys (Kojima, 1985; Lu and Wang, 2000; Wang, 2000; Mustovic et al., 2003; Fritz et al., 2005). We assume the TITW as the time during which multiple events are integrated to form a single percept of the time interval. For the interval percept, single TITWs necessarily have to include at least two events or timing signals. For discrimination, an interval percept needs to be compared with other interval percepts already kept as memory. Based on this assumption, if a sound consists of a sequence of sound segments with the ISI *longer* than this putative TITW, animals can process maximally only one acoustic event within single TITWs. Consequently, in this combination of the TITW and ISI, the ISI of the sound sequence cannot be transformed into an ISI percept. In contrast, if the TITW is long enough to include more than one consecutive timing signals, such as the repeated segments of shorter-ISI test sounds used in the present study (e.g., 33%- or 50%-ISI sounds), the timing of these segments can be integrated in single TITWs, resulting in the generation of an ISI percept.

Keeping in mind this assumption, we measured reaction times (RTs) for behavioral initiation of every animal to each of the ISI test sounds and also to the T sound used as the positive control. As shown in Figure 9, the RTs for the animal population were distributed in a relatively wide range for any ISI test sound. This variability of RTs could be interpreted as follows; if an animal successfully detects the first segment, its RT would be the minimum in this population range, but if it fails to detect the first segment but misperceives a later segment to be the onset segment, the RT would be a longer one. Therefore, it is most plausible that the minimum value in the ISI population range should correspond to the TITW for the subject. Since in the present study animals relied on memory created through training with the CS, we adopted the minimum RT for *the CS sound* (i.e., *T sound*) as the TITW of the GP. As described in Results section, RTs to the T sound were ranged from 0.6 to 2.4 s, 0.6 to 3.0 s, 0.6 to 3.2 s, and 0.4 to 3.5 s when it was played as the positive control in the respective cases of 33, 50, 150, and 200%-ISI test trials. The grand mean of these RTs was 0.55 s (*n* = 4). The ISI of the T sound (0.63 s) is *longer* than this TITW duration, meaning that *only* one *segment* of the T sound can be processed within this presumed TITW, so no ISI percept can be generated. It suggests that during training with the CS, the ISI information of the CS (i.e., T sound) may not have been used as the cue for its recognition. Rather, spectral features of the CS segments were likely to be used as the cue for its recognition. If so, it is highly possible that the first segment of any tempo-test sound could drive animals to initiate the BhRs, because the first segments of all tempo-test sounds were the same as that of the CS (or T sound) segments. In accordance with this implication, the present tempo generalization test showed no significant difference in the minimum RT as well as in the mean RT among all the test tempos used.

Although RTs were similar among different test tempos, behavioral performance varied greatly among these test tempos (compare Figure 8 and Figure 9). This variability in behavioral performance to the different tempos can be explained comprehensively by assuming that the number of timing signals to be integrated within single TITWs depends on the ISI duration of test sounds. Since the ISIs of the slower-tempo, so longer-ISI, sounds (i.e., 0.95 and 1.25 s ISIs for the 150 and 200%-ISI test sounds, respectively) are *longer* than the estimated TITW (i.e., 0.55 s), more than one segment of these test sounds were not integrated within the TITW, indicating that ISI percepts could not be generated for these longer-ISI test sounds. Indeed, animals did not discriminate the longer-ISI test sounds from the reference T sound at all, as shown in Figure 8. In contrast, when the ISI of test sounds was *shorter* than the estimated TITW, multiple segments of the test sounds were integrated within single TITWs for ISI percepts, suggesting that animals could discriminate these shorter-ISI test sounds from the T sound. Indeed, this was the case for the shorter-ISI test sounds, as shown in Figure 8. Since the ISI of the 33%-ISI test sound was 0.21 s and that of the 50%-ISI test sound was 0.32 s, three and two segments of the respective test sounds were included within the 0.55 s-long TITWs. Figure 8 shows that the shortest-ISI test sound was discriminated almost perfectly from the T sound (positive animal ratios of 1/18 and 17/18 in the 33%-ISI test and T sound trials; statistically significant) and that the 2nd shortest ISI test sound was substantially discriminated from the T sound (6/18 and 18/18 in the 50%-ISI test and T sound trials; statistically significant). It should be noted that such discrimination is possible only when animals come to notice or attend to later segment(s), meaning that the timing of recognition of the shorter-ISI test sounds would be delayed. This view should predict the temporal development of discrimination behavior in which animals initiate apparently positive approaching behavior in response to the first (or sometimes later) segment, probably based on the spectral cues, and then at a certain delay discontinue it, probably because of the perception of unfamiliar ISIs. This was in fact frequently observed for the shorter-ISI test sounds, as shown in the 3rd scene of Video 3.

## Concluding remarks

If the minimum RT to the CS reflects the TITW for processing and coding of timing information, it is possible to comprehensively explain the behavioral variability to the test sounds different in tempo. However, alternative interpretations are also possible. Findings from the tempo discrimination test suggest that the GP may have an inherent, asymmetrical disposition in tempo perception, implying that faster tempos of natural sounds might be more meaningful for the survival or social behavior of this species, such as approaching sounds produced by predators in their final stage of attack. Such an evolutional aspect of the sound processing needs further investigation.

The GP is a more preferable animal model than rats and mice for research of the cochlear functions and implant, since its cochlea is of an easily accessible size (Pfingst et al., 2011; Agterberg and Versnel, 2014) and the frequency rage of its audiogram overlaps, especially for the lower part, with that of the human extensively (Heffner et al., 2001). Traditionally, hearing capabilities of GPs with cochleae manipulated pharmacologically or mechanically have been evaluated on the basis of discrimination of the tonal quality or sound level (Prosen et al., 1981; Nicol et al., 1992; Kang et al., 2010; Pfingst et al., 2011; Dehmel et al., 2012; Agterberg and Versnel, 2014; Heeringa et al., 2014). The present findings showing that the GP has abilities to discriminate sounds differing in spectral and fine temporal structures may facilitate the availability of this animal species for the evaluation of how well the perception of sounds recovered after experimental manipulations. The present study also showed the possibility that the GP was able to recognize acoustic tempos and discriminate between sounds with faster and slower tempos. It is interesting to see how the stereotyped contact behavior evoked by the purr call of GPs (Harper, 1976) would be affected, if the ISI of repetitive segments of this call is modified.

## Author contributions

HO designed the study and made behavioral experiment, JH made the statistical analyses, and HO and JH wrote the paper.

### Conflict of interest statement

The authors declare that the research was conducted in the absence of any commercial or financial relationships that could be construed as a potential conflict of interest.
