# Supplementary material for: Recognition of Modified Conditioning Sounds by Competitively Trained Guinea Pigs
Source: Front Behav Neurosci. 2016 Jan 26;9:373. doi: 10.3389/fnbeh.2015.00373 (PMC4726754; doi:10.3389/fnbeh.2015.00373)
Supplement: Supplementary file 4 [file DataSheet1.DOCX]

**Supplementary Material**

Video 1: Conflict between pair-trained guinea pigs in the early stage of the competitive training. Soon after the onset of the conditioning sound (CS), two guinea pigs start to approach the food container. One of them blocks the other to extend his nose to the food hopper, in this particular case, or to approach the pellet saucer, in general, during the sound-on period. The conflict usually continues for a short while even after the feeding, as shown here. CS is a sequence of repeating noise-like footstep-sound segments. Behavior of animal(s), recorded at 3 different angles, is presented in 3 separate windows. Timings of the head/body movements of animal(s), feeding timing, and sounds are shown on a single chart in the lower-right window. Brief silent gaps heard during playback of the video are artifacts generated during editing process.

Video 2: Typical behaviors of a training-completed guinea pig in the test trial. As shown in the first scene, in response to the target sound ("step sound", T) that has been used as the conditioning sound (CS) during training, the subject evokes quick and busy movements such as head swaying and neck extension and/or circling locomotion around the saucer (not for this particular subject), during the sound-on period (behavioral reaction, BhR) and typically continue it till the time when a pellet is fed (positive response). In contrast, in response to non-target (NT)　sounds,　subjects initiate no typical BhRs, usually keeping stationary as typically shown in the 3rd scene of this movie (metal plate-hitting sound used in this particular trial, "metal plate"). The subject is keeping stationary by the saucer as he is exposed to NT sounds. Also, between the above two scenes, note the scene showing typical behaviors in response to one of the temporally modified test sounds ("segment-reversed (sR)", segR). Animal's behavior, recorded at 3 different angles, is presented in 3 different windows. Lower-right window illustrates a chart of live recording including, from top to bottom, timings of head/body movements, timing of the feeding, sounds monitored by an in-room microphone, and sounds saved in a computer and played through in-room speakers.

Video 3: Behavior to the test sounds that are comprised of a sequence of sound segments whose intervals are changed relative to that of the target (T) sound. Behaviors of an animal to (1) the T sound (100% interval), (2) a longer-interval test sound whose intersegment interval (ISI) is doubled (200% interval), and (3) a shorter-interval test sound that has the ISI shortened to one third of that of the T sound (33% interval) are shown in this order. Note that the distinct behavioral reactions (BhRs) such as quick head swaying and/or neck extension are evoked soon after the onset of the T sound and also 200%-ISI test sound, while they are initiated but soon discontinued during the sound-on period for the 33%-ISI test sound. The behavior to the 33%-ISI test sound is assessed to be negative (so discriminative) because of the break off of the once-initiated BhRs, indicating that the animal perceives this test sound to be different from the T sound.

Audio 1. Sounds used for training will be played in order of the conditioning sound (footstep, T), handclap (NT1), hitting plastic cage (NT2), hitting metal (NT3), scratching metal mesh (NT4) and jingling keys (NT5).

Audio 2. Single segments from sounds used for the spectral modification test will be played in order of the conditioning sound (T) and each of pseudo-target (PsY) sounds in which a frequency range centered (in octave) at either 0.6, 1.8, 2.8 or 4.9 kHz is separately removed from the bandwidth of the T sound. Amplitudes of these PsT sounds are adjusted to that of the T sound before playback in test sessions.

Audio 3. Single segments from sounds used for the fine temporal modification test will be played in order of the conditioning sound (T), segR, ONrev, and ONcut. See test for details.

Audio 4. Single segments from sounds used for the tempo modification test will be played in order of pseudo-target (PsT) sounds with the samller magnitude of modification on intersegment interval. PsT100% is the T sound used for conditioning.
